# Cobalt chloride compromises transepithelial barrier properties of CaCo-2 BBe human gastrointestinal epithelial cell layers

**DOI:** 10.1186/s12876-017-0731-5

**Published:** 2018-01-05

**Authors:** K. M. DiGuilio, M. C. Valenzano, E. Rybakovsky, J. M. Mullin

**Affiliations:** 1 Lankenau Institute for Medical Research, 100 Lancaster Avenue, Wynnewood, PA 19096 USA; 2 Division of Gastroenterology, Lankenau Medical Center, Wynnewood, PA 19096 USA; 3 Present Address: Philadelphia College of Osteopathic Medicine, 4170 City Avenue, Philadelphia, PA 19131 USA

**Keywords:** CaCo-2, Tight junction, Claudin, Cobalt, HIF-1, Occludin, Transepithelial, Paracellular, Barrier function

## Abstract

**Background:**

Elevation of the transcription factor HIF-1 is a prominent mediator of not only processes that accompany hypoxia, but also the tumor microenvironment and tissue regeneration. This study uses mediators of “chemical hypoxia” to ask the question whether HIF-1α elevation in a healthy epithelial cell layer leads to leakiness in its tight junctional seals.

**Methods:**

Transepithelial electrical resistance and transepithelial diffusion of ^14^C–D-mannitol and other radiolabeled probes are used as indicators of transepithelial barrier function of CaCo-2 BBe human gastrointestinal epithelial cell layers cultured on permeable supports. Western immunoblot analyses of integral tight junctional proteins (occludin and claudins) are used as further indicators of barrier function change.

**Results:**

Cobalt, an inhibitor of the prolyl hydroxylase enzymes governing HIF-1α breakdown in the cell, induces transepithelial leakiness in CaCo-2 BBe cell layers in a time and concentration-dependent manner. This increased leakiness is accompanied by significant changes in certain specific integral tight junctional (TJ) proteins such as a decreased level of occludin and increased level of claudin-5. Similar results regarding barrier function compromise also occur with other chemical inhibitors of HIF-1α breakdown, namely ciclopiroxolamine (CPX) and dimethyloxalylglycine (DMOG). The increased leak is manifested by both decreased transepithelial electrical resistance (R_t_) and increased paracellular diffusion of D-mannitol (J_m_). The induced transepithelial leak shows significant size selectivity, consistent with induced effects on TJ permeability. Less-differentiated cell layers were significantly more affected than well-differentiated cell layers regarding induced transepithelial leak. A genetically modified CaCo-2 variant with reduced levels of HIF-1β, showed reduced transepithelial leak in response to cobalt exposure, further indicating that elevation of HIF-1α levels induced by agents of “chemical hypoxia” is responsible for the compromised barrier function of the CaCo-2 BBe cell layers.

**Conclusions:**

Exposure to inducers of chemical hypoxia elevated HIF-1α levels and increased transepithelial leak. The degree of epithelial differentiation has significant effects on this action, possibly explaining the varying effects of HIF-1 modulation in epithelial and endothelial barrier function in different physiological and pathophysiological conditions.

**Electronic supplementary material:**

The online version of this article (doi: 10.1186/s12876-017-0731-5) contains supplementary material, which is available to authorized users.

## Background

The HIF-1α (hypoxia-inducible factor 1α) monomer in combination with the HIF-1β monomer, constitute in part the HIF-1 heterodimer transcription factor that is responsible for transcriptional regulation of a wide array of genes including those that favor the creation of a (precancerous) tumor microenvironment and survival in hypoxia in general. Increased levels of HIF-1α in the cytosol (e.g., by inhibition of HIF-1α degradation) result in elevated levels of the HIF-1 heterodimer transcriptional factor being formed in the nucleus where its regulation of gene transcription then occurs. Genes whose transcription is increased by HIF-1 include glucose transport proteins, key glycolytic enzymes, Vascular Endothelial Growth Factor (VEGF), Transforming Growth Factor-β1 (TGF-β1), TWIST, c-Met, lysyl oxidase (LOX), and the anti-apoptotic gene, MCL-1. Increased transcription of these genes favors cancer development and metastasis. Regulation of HIF-1 levels has received great attention not only in the field of cancer biology but also in regenerative medicine, autoimmune diseases and hypoxia-related conditions generally [1–5].

Cobalt and other mediators of “chemical hypoxia” such as desferrioxamine (DFO), ciclopirox olamine (CPX), and dimethyloxallylglycine (DMOG) have in common an increase in the cellular content of the protein HIF-1α, achieved in part by these compounds’ interference with the prolyl hydroxylase enzyme responsible for initiating the degradation of HIF-1α [6]. Cobalt therefore typically causes increased levels of HIF-1α in cells because degradation of HIF-1α is inhibited while its synthesis is ongoing.

In this current study we sought to ask one question: Does the treatment of intact, functional epithelial cell layer barriers with cobalt weaken, strengthen or have no effect on epithelial barrier function, using a highly investigated in vitro model of human intestinal epithelia, the CaCo-2 BBe cell line [7]. The published literature presents a somewhat bifurcated viewpoint on the issue of HIF-1 and epithelial/endothelial barrier function. There is a great deal of literature using mainly brain capillary endothelial cell culture models that indicates that compounds/conditions that increase HIF-1α levels cause leakiness in tight junctions and consequently cellular barriers [8]. On the other hand, other researchers, using mainly epithelial cell culture and rodent gastrointestinal tissue models have observed that compounds/conditions that increase cellular levels of HIF-1α are supportive of barrier recovery of intestinal cell layers from colitis-like experimental protocols [9]. While it is true that one has quite different cell models here (endothelial vs epithelial), these are two seemingly very opposed sets of findings. However, it needs to be kept in mind that the two disparate findings emerge from different starting conditions — those studies showing HIF-1 to be barrier *compromising* start from a fully functional, intact cell layer barrier. The studies showing HIF-1 to be barrier-enhancing start from an already compromised epithelial barrier that is engaged in repair processes to reinstitute barrier function. We believe this distinction is key to the apparent qualitative difference in outcomes, and we show data examining cobalt’s effects on cell layers at different degrees of differentiation that suggest that this is indeed the case.

## Methods

### Cell culture

The CaCo-2 BBe cell culture, an epithelial cell line derived from human colon adenocarcinoma [7], was obtained from ATCC and was used between passages 52 and 70. Upon confluence, cells were passaged on a weekly basis by trypsinizination (0.25% trypsin and 2.2 mM EDTA [Corning Cellgro]) and were seeded at 5 × 10^5^ cells/Falcon 75-cm2 culture flask with 25 ml of Dulbecco-s Modified MEM (25 mM glucose) (Minimum Essential Medium) (Corning Cellgro) supplemented with 2 mM L-Glutamine (Corning Cellgro), 1% Non Essential Amino Acids (Corning Cellgro), 1 mM Sodium Pyruvate (Corning Cellgro) and 10% defined fetal bovine serum (HyClone). Cultures were incubated at 37 °C in 95% air/5% CO2 atmosphere.

### Transepithelial permeability measurements

Cells were seeded into sterile Millicell polycarbonate (PCF) permeable supports (30 mm diameter with 0.4 μm pore size) (Millipore, Inc.) on day 0 at a seeding density of 5 × 10^5^ cells/insert. This is approximately 50% of confluent density. Three or 4 sterile Millicell PCF inserts were placed into a 100 mm petri dish. On day 1, all cell layers were refed (2 ml apical/15 ml basal-lateral) with control medium containing 50 U/ml penicillin and 50 μgms/ml streptomycin, followed by refeedings every 2–3 days until treatment, then followed by electrophysiological measurements and radiotracer flux studies.

On the day of transepithelial experiments, the cell layers were refed with fresh control medium and allowed to incubate at 37 °C for 1.5 h prior to electrophysiological readings. All electrophysiological measurements were made in culture medium. Transepithelial potential difference was measured at 37 °C using 1 M NaCl salt bridges in series with calomel electrodes. Transepithelial electrical resistance (R_t_) was measured at room temperature using 1 s, 40 μamp direct current pulses (through 1 M NaCl salt bridges in series with Ag/AgCl electrodes) in a custom-made Lexan chamber designed to accept the Millicells, and calculated using Ohm’s law. Current-passing and voltage-measuring salt bridges were positioned above and below the center point of the cell layers. As soon as electrical measurements were completed, the basal-lateral medium was aspirated and replaced with 15 ml of medium containing 0.1 mM, 0.1 μCi/ml ^14^C–D-mannitol (Perkin-Elmer, Boston, MA) or other radiolabeled probe, and incubated at 37 °C. Triplicate basal-lateral medium samples (50 μl) were taken for liquid scintillation counting (LSC) for specific activity determination. Duplicate samples (100 μl) were taken from the apical side at 60 min for LSC to determine flux rates. The flux rate (J_m_) (in cpm/min/cm^2^ and picomoles/min/cm^2^) was calculated for the ^14^C–D-mannitol diffusing across the cell layer. Flux rate studies involving radiolabeled lactulose and polyethylene glycol also utilized 0.1 mM total concentrations, and flux rates were determined similarly.

Due to our procedure of seeding Millicell PCFs at approximately 50% of confluent density, a 3-day (post seeding) culture is 2-days-post-confluence. A 7-day (post seeding) culture is 6-days-post-confluemnce, and so on. In this publication, cultures are defined by days-post-seeding.

### Analyses of tight Junctional proteins

Following transepithelial resistance or transepithelial mannitol diffusion studies, cell layers on Millicell PCF membranes were washed 5X in 4 °C phosphate-buffered saline (PBS) and then harvested by carefully scraping into lysis buffer, followed by sonication and ultra-centrifugation. Samples of these fractions were analyzed by PAGE using a 4–20% gradient Novex Tris-glycine gel at 125 V for 1 h 45 min. 4–12% Novex Tris-glycine gels were used for HIF-1α and HIF-1β determinations. Precision Plus Kaleidoscope Protein Standards (Biorad, Inc.) were also included in each gel. Proteins were transferred at 30 V for 2 h from the gel to a PVDF membrane. The membranes were then stained with MemCode™ reversible protein stain, and densitometry measured. Blots were then washed 3 times with PBS-T (0.3% Tween-20) for 10 min each and blocked with 5% milk/PBS-T for 1 h at RT. Membranes were incubated with the specific primary antibody (anti-claudin-5, anti-claudin-7, anti-occludin [Life Technologies]) at 1.0 μg/ml in 5% milk/PBS-T at 4 °C. For claudin-5 this incubation was for overnight, followed by 2 h at RT. For occludin and claudin-7 there was only a 2 h incubation with the primary antibody at room temperature. The membranes were washed with PBS-T 3X for 10 min each, then incubated with secondary antibody (rabbit anti-mouse or goat anti-rabbit IgG labeled with horseradish peroxidase [Southern Biotechnology]) for 1 h at RT. Membranes were washed with PBS-T (4X for 10 min each), then treated for 1 min with Western Lightning-ECL chemiluminescence reagents (Perkin Elmer). The membranes were then exposed to HyBlot CL autoradiography film (Denville Scientific), which was developed in a Kodak M35A X-OMAT processor. Band densities were quantified by densitometry. Band densities are reported either as absolute values or against normalized averages of corresponding control band densities. All bands are also normalized to a densitometric determination of total protein in that specific immunoblot as visualized by the MemCode reversible staining kit (Thermo Fisher, Inc.). Full length Western immunoblots for all tight junctional proteins (as well as HIF-1a and HIF-1b) are shown in Additional files 1, 2, 3, 4 and 5. 

### Cell viability

The Cytotox96 cell viability assay was a product of the Promega Corporation. This is a colorimetric-based assay that examines lactate dehydroenase release from non-viable cells.

### Statistics

For electrophysiology, radiotracer flux and protein chemistry studies, Co^++^-treated cell samples (and other experimental treatments) were compared against appropriate matched controls within the same experiment. All data are expressed as the mean ± standard error of the mean (SEM) with the number of replicates provided for each set of studies. Differences between means are evaluated by two-tailed Student’s t tests for two groups, or by one-way analysis of variance (ANOVA) followed by Tukey’s post-hoc testing for multiple comparisons.

### Reagents

Cobalt chloride hexahydrate was a product of Sigma Chemical Co. and was used as a 100 mM stock solution in water. Ciclopirox olamine was also purchased from Sigma and was used as a 250 mM stock in methanol. Dimethyloxalylglycine was used as a 100 mM stock solution in water and was obtained from EMD Millipore. All culture medium and associated reagents were a product of Corning Cellgro (Mediatech, Inc). The fetal bovine serum used in the culture medium was a product of HyClone (Thermo Fisher Corp.).

## Results

**Fig. 1:**
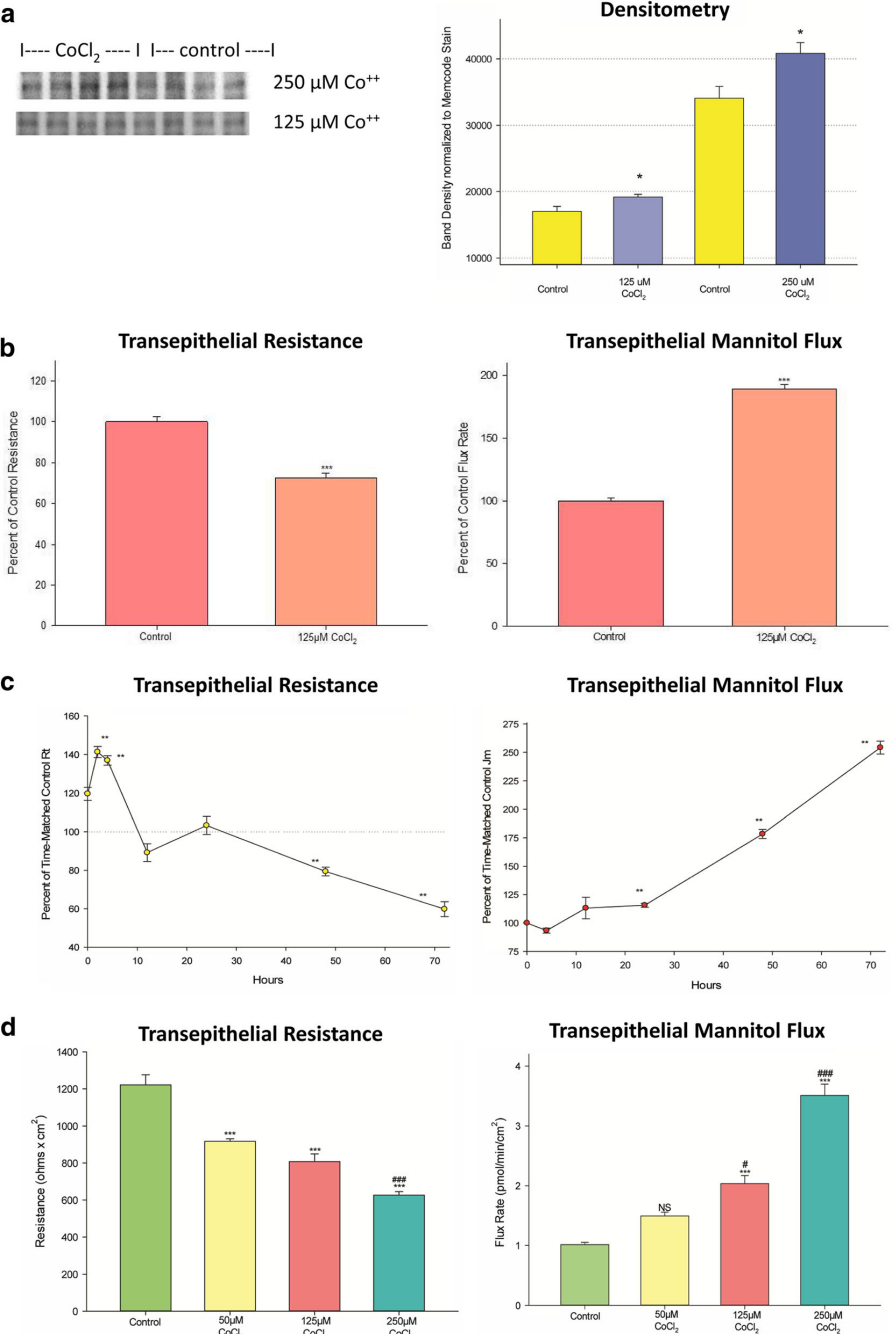
The effect of cobalt chloride on CaCo-2 BBe transepithelial electrical resistance and transepithelial flux of ^14^C–D-mannitol. **a** Western immunoblot data showing the significant increase of HIF-1α levels in whole cell lysates of 4 individual, 7-day post-confluent CaCo-2 BBe cell layers exposed to 125 and 250 μM CoCl_2_ (or vehicle control) for 48 h. **b** The effect of Co^++^ on barrier function. Seven-day post-confluent CaCo-2 BBe cell layers were refed in control medium or medium containing 125 μM CoCl_2_ on the apical and basal-lateral sides 48 h prior to electrical measurements. Data shown represent the percent of control resistance expressed as the mean ± SEM of 20 cell layers per condition. After electrical measurements, radiotracer flux studies with 0.1 mmol/L, 0.1 μCi/mL ^14^C–D mannitol were performed on the same CaCo-2 BBe cell layers, as described in Materials and Methods. Data shown represent the percent of control mannitol flux rate and are expressed as the mean ± SEM of 20 cell layers per condition. ***P < 0.001 vs control. (Student’s t test, two-tailed). **c** The time course of changes in transepithelial electrical resistance and transepithelial mannitol flux resulting from CoCl_2_ exposure. Seven-day post-seeding CaCo-2 BBe cell layers on Millicell PCF filters were refed in control medium or medium containing 125 μM CoCl_2_ (apical and basal-lateral compartments) and transepithelial electrical resistance was recorded at 2, 4, 12, 24, 48, and 72 h (different cell layers were used for each designated time point). Data shown represent the percentage of time-matched resistance (relative to vehicle-treated control cell layers) and are expressed as the mean ± SEM of 4 cell layers per condition. After electrical measurements, radiotracer flux studies with 0.1 mmol/L, 0.1 μCi/mL ^14^C–D mannitol were performed on the same CaCo-2 BBe cell layers. **P < 0.01 (two tailed Student’s t test vs time-zero cell layers). **d** The effect of increasing concentrations of CoCl_2_ on CaCo-2 BBe transepithelial electrical resistance and transepithelial flux of ^14^C–D-mannitol. Seven-day post-seeding CaCo-2 BBe cell layers on Millicell PCF filters were refed in control medium or medium containing 50 μM, 125 μM, or 250 μM CoCl_2_ 48 h prior to electrical measurements (apical and basal-lateral compartments). Data shown represent the mean ± SEM of 4 cell layers per condition. After electrical measurements, radiotracer flux studies with 0.1 mmol/L, 0.1 μCi/mL ^14^C–D mannitol were performed on the same CaCo-2 BBe cell layers used for resistance studies. NS indicates non significance vs control. #*P* < 0.05 vs 50 μM CoCl_2_; ###*P* < 0.001 vs 50 μM CoCl_2_; ***P < 0.001 vs control. (one-way ANOVA followed by Tukey’s post hoc testing)

Exposure of CaCo-2 BBe cell layers to 125 μM CoCl_2_ on both cell surfaces for 48 h resulted in a small but significant elevation of HIF-1α (Fig. 1a), along with a highly significant (30%) decrease in R_t_ and a corresponding simultaneous increase in transepithelial paracellular diffusion (leak) of ^14^C–D-mannitol (J_m_) by nearly 100% (Fig. 1b). 250 μM CoCl_2_ produced a greater elevation of HIF-1α (Fig. 1a). In evaluating the time course of this effect of Co^++^ on CaCo-2 BBe barrier function, cell layers were exposed to 125 μM Co^++^ on both cell surfaces for varying exposure periods out to 72 h, followed by measurement of R_t_ and J_m_. As shown in Fig. 1c, the transepithelial leakiness that ensues from Co^++^ exposure requires 48 h to be detectable by R_t_ and J_m_ measurements. Decreased transepithelial leak — evidenced especially by increased R_t_ — may be occurring at the earliest time points (2 to 6 h) that were measured. R_t_ decreased to 60% of time-matched controls, and J_m_ increased to 250% of time-matched controls by 72 h of exposure. The concentration dependence of the Co^++^ effect on R_t_ and J_m_ is shown in the graded effects of increasing Co^++^ concentrations seen in Fig. 1d. 125 μM Co^++^ showed statistically significant effects on both R_t_ and J_m_ after 48 h of exposure, whereas 50 μM Co^++^ had a significant effect on R_t_ but not J_m_. 250 μM Co^++^ decreased R_t_ by nearly 50% of control levels, while simultaneously increasing J_m_ to almost 400% of control levels.

**Fig. 2:**
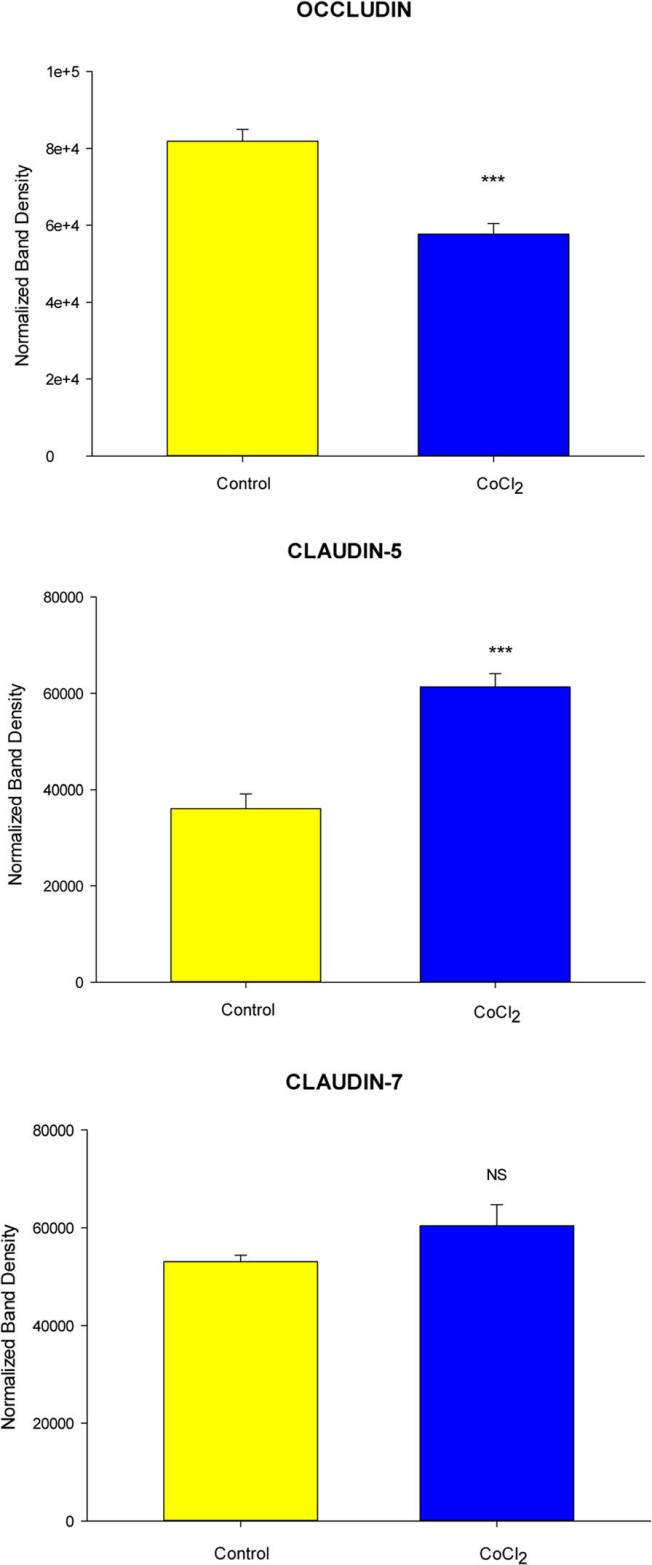
Effect of 125 μM CoCl_2_ for 48 h on the abundance of 3 integral tight junction proteins of CaCo-2 BBe cell layers. Seven-day cell layers were harvested from Millicell PCF membranes, and whole cell lysates prepared as described in Methods and Materials. Results shown represent densitometry of western immunoblots expressed as the mean ± SEM of 4 cell layers. *** *P* < 0.001, Student’s t test, two-tailed. NS: not significant

This Co^++^-induced compromise in barrier function occurred with induced changes in individual tight junctional (TJ) proteins. Occludin was decreased by over 30%, whereas claudin-5 was increased by almost 80%. No significant change occurred in the content of claudin-7 (Fig. 2). Cell layers exhibiting decreased R_t_ showed no significantly increased cell cytotoxicity as a result of 48 h exposure to 125 or 250 μM Co^++^, as determined by a lactate dehydrogenase-based viability assay (data not shown). The 125 μM Co^++^ condition showed only a 5% increase in LDH release (compared to vehicle control), and the 250 μM condition showed only a 4% increase in LDH release. Neither were statistically significant.

**Fig. 3:**
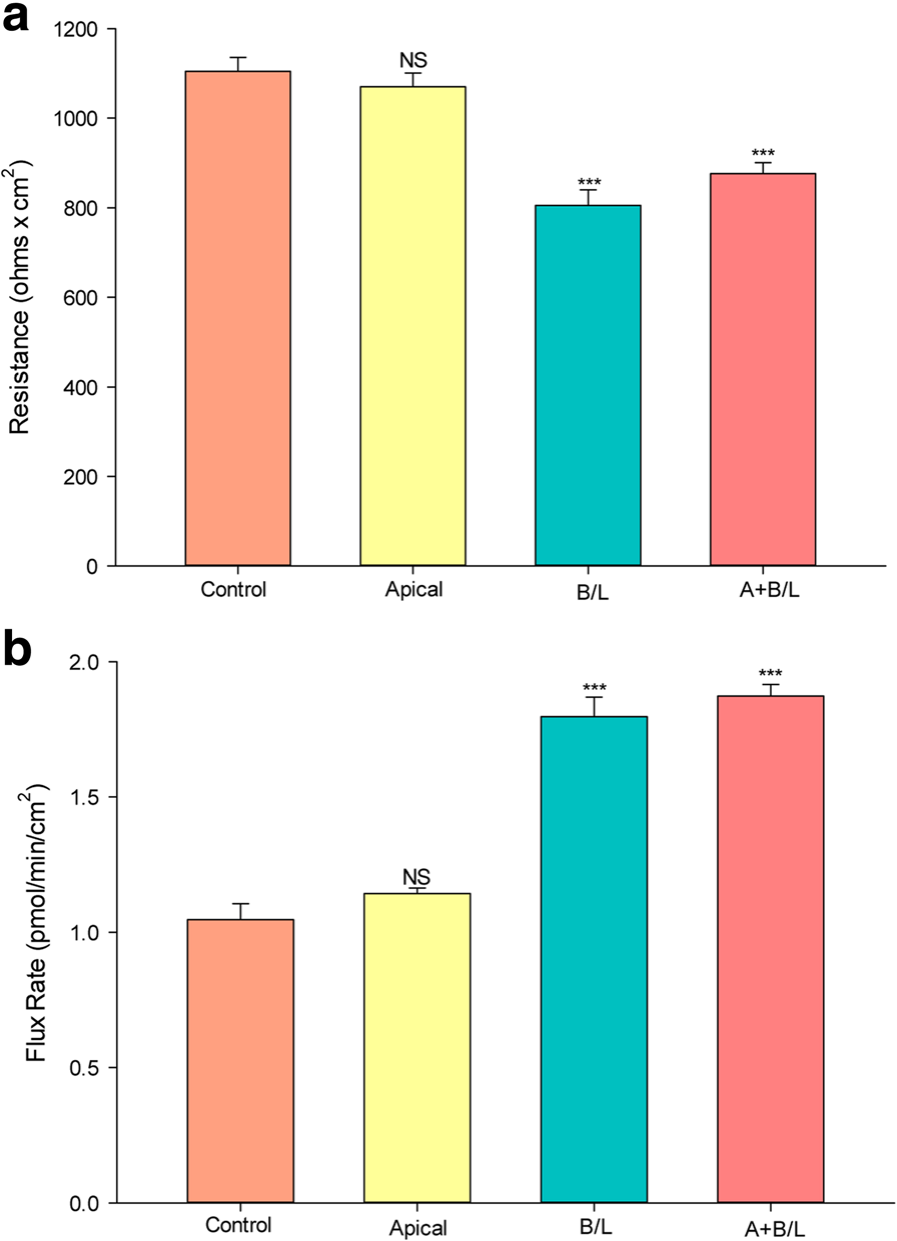
Sidedness of the CoCl_2_ effect on CaCo-2 BBe transepithelial electrical resistance and transepithelial flux of ^14^C–D-mannitol. **a** Seven-day post-confluent CaCo-2 BBe cell layers on Millicell PCF polycarbonate filters were refed in control medium or medium containing 125 μM CoCl_2_ on the apical-only, basal-lateral-only, or apical and basal-lateral compartments, 48 h prior to electrical measurements. Data shown represent the mean resistance ± SEM of 4 cell layers per condition (3 cell layers used in apical application). **b** After electrical measurements, radiotracer flux studies with 0.1 mmol/L, 0.1 μCi/mL ^14^C–D mannitol were performed on the same CaCo-2 BBe cell layers represented in A, as described in Materials and Methods. Data represent the mean flux rate ± SEM of 4 cell layers per condition. NS indicates non significance. ***P < 0.001 vs control (one-way ANOVA followed by Tukey’s post hoc testing)

Addressing the issue of the epithelial surface from which Co^++^ was able to produce this effect, the experiments of Fig. 1 were repeated, but now adding CoCl_2_ to culture medium bathing one or the other cell surface or both. As shown in Fig. 3, this effect of Co^++^ was mediated specifically by Co^++^ exposure to the basal-lateral cell surface. Co^++^ exposure to the apical cell surface produced no significant effect on either R_t_ or J_m_. Co^++^ exposure to both cell surfaces simultaneously, produced the same effect as Co^++^ exposure to only the basal-lateral cell surface.

**Fig. 4:**
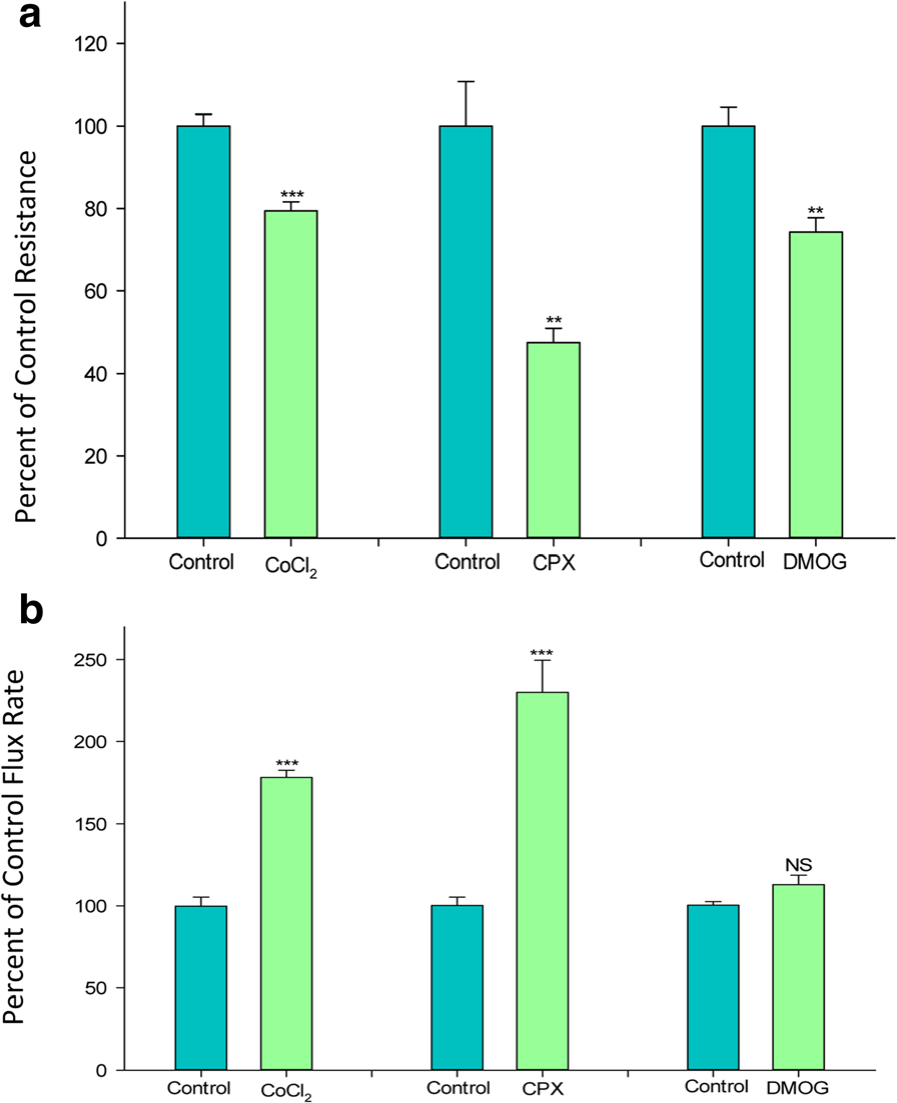
The effect of the different HIF-1α up-regulators (cobalt chloride, DMOG-Dimethyloxalylglycine, CPX- Ciclopirox olamine) on CaCo-2 BBe transepithelial electrical resistance and transepithelial flux of ^14^C–D-mannitol. **a** Seven-day post-confluent CaCo-2 BBe cell layers on Millicell PCF filters were refed in control medium or medium containing 125 μM CoCl_2_ for 48 h, 15 μM CPX for 48 h, or 1.0 mM DMOG for 72 h (apical and basal-lateral compartments) prior to electrical measurements. Data shown represent the percent of control resistance and is expressed as the mean ± SEM of 4 cell layers per condition. **b** After electrical measurements, radiotracer flux studies with 0.1 mmol/L, 0.1 μCi/mL ^14^C–D mannitol were performed on the same CaCo-2 BBe cell layers represented in A. NS indicates non significance. **P < 0.01 and ***P < 0.001 vs respective controls (Student’s t test, two-tailed)

Other “chemical hypoxia” agents known to elevate HIF-1α, namely ciclopirox olamine (CPX) and dimethyloxalylglycine (DMOG), were examined (at concentrations reported to elevate HIF-1α) for their effects on transepithelial barrier function. As shown in Fig. 4, like Co^++^, CPX (at 15 μM) both significantly decreased R_t_ and increased J_m_ in a 48 h exposure. DMOG (1 mM) significantly decreased R_t_ but the increase it produced in J_m_ was not statistically significant.

**Fig. 5:**
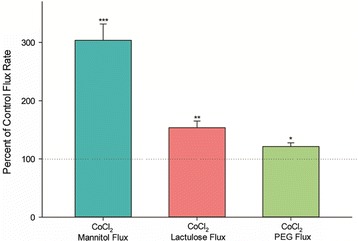
The effect of CoCl_2_ on transepithelial flux of ^14^C–D-mannitol, ^3^H–lactulose, and ^14^C–polyethylene glycol across CaCo-2 BBe cell layers. Seven-day post-confluent CaCo-2 BBe cell layers on Millicell PCF filters were refed in control medium or medium containing 125 μM CoCl_2_ (basal-lateral compartment) 72 h prior to radiotracer flux studies. These studies were performed using 0.1 mmol/L, 0.1 μCi/mL ^14^C–D-mannitol; 0.1 mmol/L, 0.25 μCi/mL ^3^H–lactulose; and 0.1 mmol/L, 0.1 μCi/mL ^14^C–polyethylene glycol as described in Materials and Methods. Data shown represent the percent of control flux rate and is expressed as the mean ± SEM of 4 cell layers per condition. *P < 0.05, **P < 0.01, ***P < 0.001 vs respective controls (Student’s t test, two-tailed)

In conditions where Co^++^ produced a 200% increase in J_m_ (125 μM, 72 h), we examined whether there was a size-selective “sieving” characteristic to the transepithelial leak by observing the transepithelial paracellular diffusion of different-sized molecular probes. Unlike the 200% increase seen for ^14^C–D-mannitol (182 Da) leak in the presence of Co^++^, the larger paracellular probe, ^3^H–lactulose (350 Da) showed only a 45% increase in transepithelial leak (Fig. 5). All flux studies were performed using 0.1 mM concentrations of the radiolabeled probe. The still larger paracellular probe, ^14^C–polyethyleneglycol (4000 Da) showed only a 20% increase. This suggests that the transepithelial leak produced by Co^++^ possesses sieving characteristics on, at least, a size basis. These comparative flux studies were performed after an extended 72 h incubation with Co^++^. This longer incubation underscores this point that the effect of Co^++^ on transepithelial leakiness still results in a transepithelial leak that manifests size selectivity. Whether such leak demonstrates charge selectivity has yet to be determined.

**Fig. 6:**
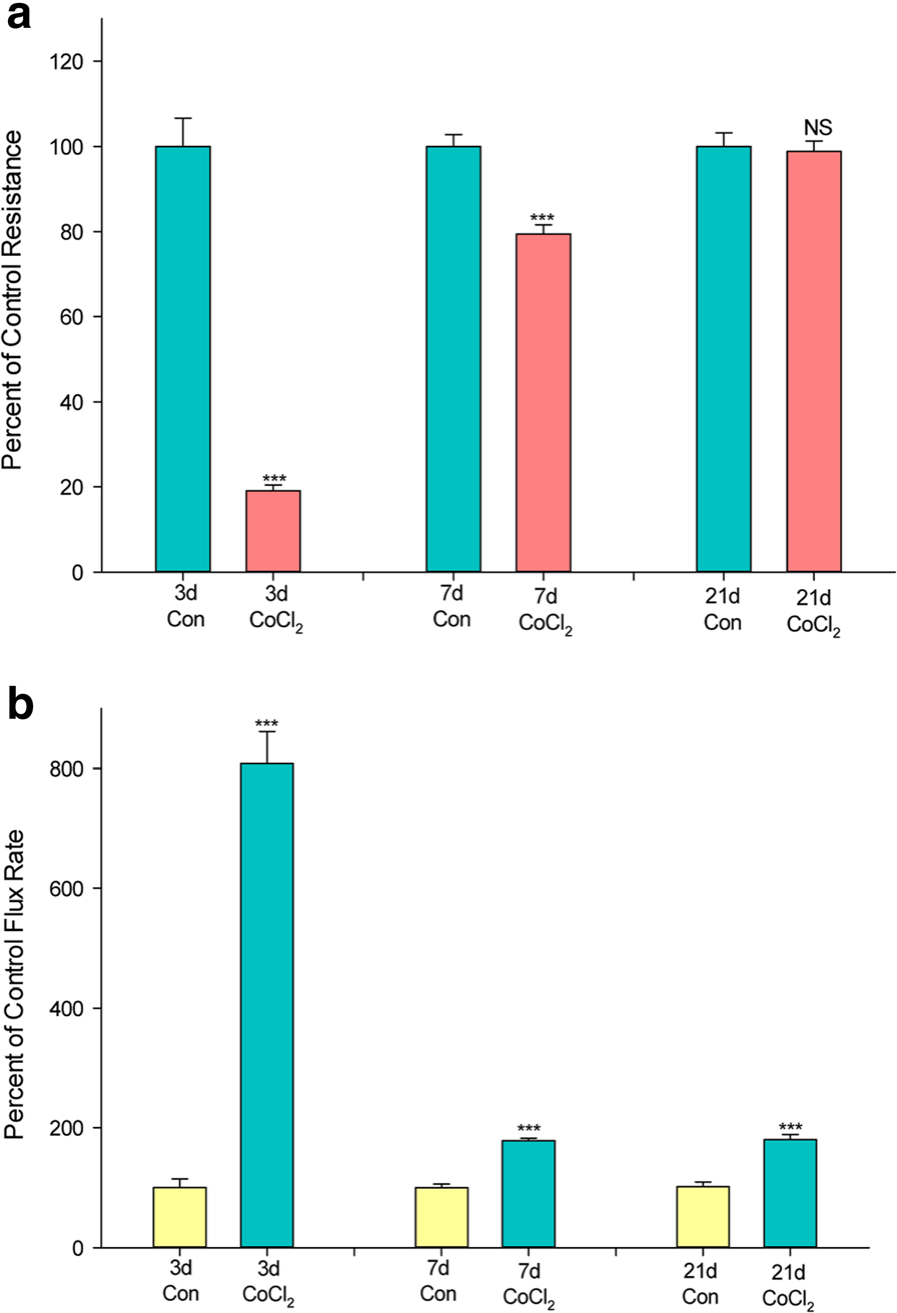
The effect of CoCl_2_ on CaCo-2 BBe transepithelial electrical resistance and transepithelial flux of ^14^C–D-mannitol as a function of the differentiation state of the cell layer. **a** Three-day, 7-day, and 21-day CaCo-2 BBe cell layers were treated on the apical and basal-lateral sides with 125 μM CoCl_2_ for 48 h before electrical measurements. Data shown represent the percent of control resistance and are expressed as the mean ± SEM of 8 cell layers per condition in the 3-day and 21-day experiments and 4 cell layers per condition in the 7-day experiment. **b** After electrical measurements, radiotracer flux studies with 0.1 mmol/L, 0.1 μCi/mL ^14^C–D mannitol were performed on the same CaCo-2 BBe cell layers represented in A. NS indicates non significance vs control. ***P < 0.001 vs respective controls (Student’s t test, two-tailed)

CaCo-2 BBe epithelia are known to require significant time after reaching confluence to achieve maximal states of differentiation [10]. In prior work we demonstrated in fact that TJ complexes change as a function of time post confluence [11]. Seven-day cultures have attained near-maximal transepithelial electrical resistance, even though other differentiated features, such as surface membrane hydrolases, SGLT transporters, etc., may require a full 21 days post seeding for maximal expression. We therefore analyzed whether the transepithelial leak in CaCo-2 BBe cell layers produced by Co^++^ is affected by the state of CaCo-2 BBe differentiation. As shown in Fig. 6, less differentiated (3-day) CaCo-2 BBe cell layers were significantly more affected by Co^++^ than more highly differentiated (21 day post seeding) cell layers, in terms of transepithelial leak. A massive 8-fold increase in transepithelial mannitol leak was induced by cobalt in 3-day old cell layers, as opposed to only a 100% increase in 21-day-post-seeding cell layers. An 80% decrease in R_t_ was induced by Co^++^ in 3-day cell layers, whereas Co^++^ had no significant effect on the R_t_ of 21-day cell layers. Control 3-day cell layers had an average resistance of approximately 225 Ω x cm^2^, whereas 21-day cell layers manifested average resistances over 1000 Ω x cm^2^.

**Fig. 7:**
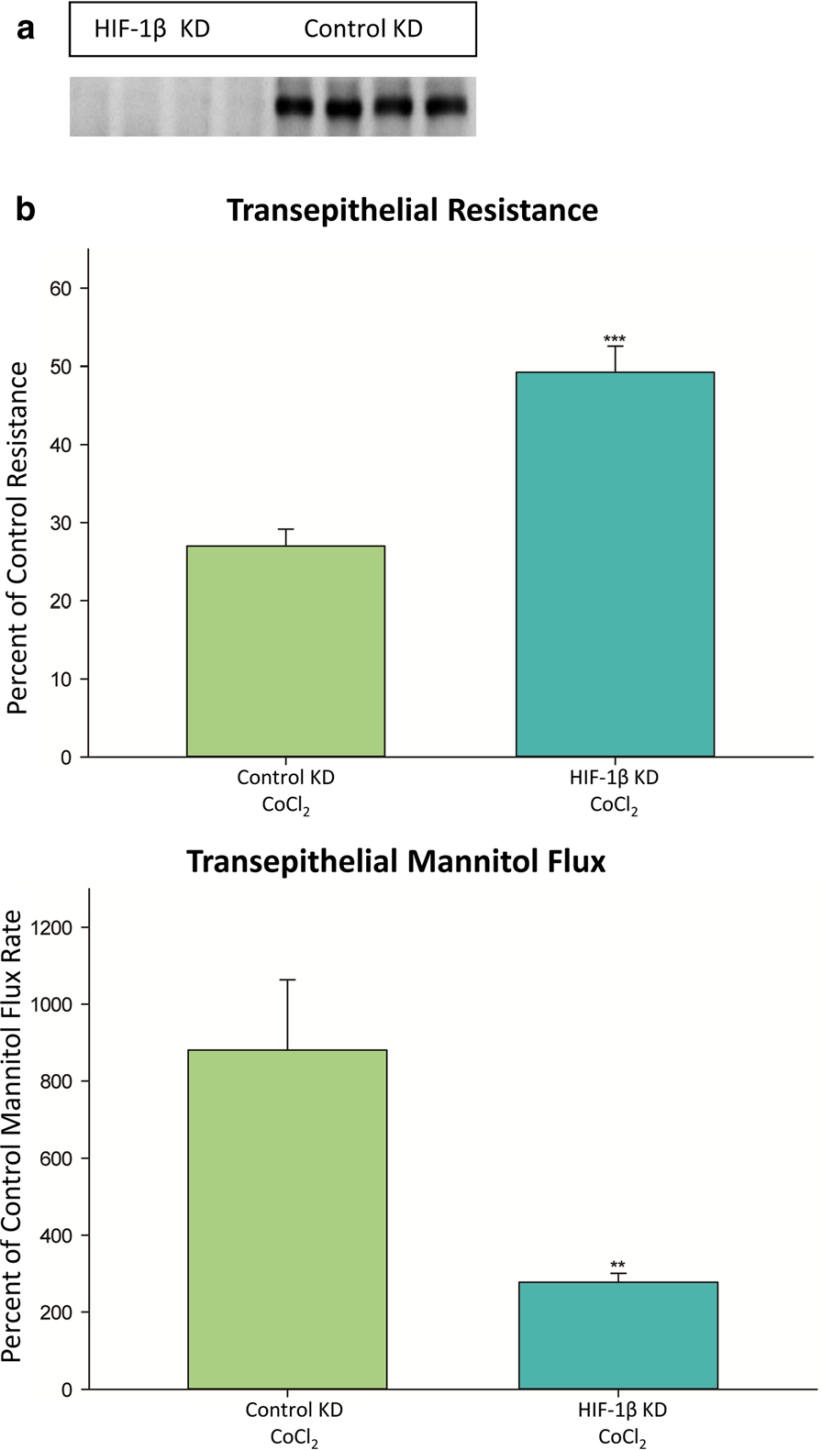
The effect of CoCl_2_ on transepithelial electrical resistance and transepithelial flux of ^14^C–D-mannitol of HIF-1β knockdown CaCo-2 cell layers. **a** Western immunoblot data showing the levels of HIF-1β protein in whole cell lysates of 4 control CaCo-2 cell layers (lanes 5–8) vs 4 HIF-1β knockdown cell layers (lanes 1–4). **b** Three-day-post-seeding control cell layers and 3 HIF-1β knockdown CaCo-2 cell layers (also 3-day-post-seeding) on Millicell PCF polycarbonate filters were refed in control medium or medium containing 125 μM CoCl_2_ on the apical and basal-lateral sides, 48 h prior to electrical measurements. Data shown represent the percent of respective control resistance and are expressed as the mean ± SEM of 8 cell layers per condition. After electrical measurements, radiotracer flux studies with 0.1 mmol/L, 0.1 μCi/mL ^14^C–D mannitol were performed on the same CaCo-2 cell layers for which resistance measurements were performed. Data shown represent the percent of respective control mannitol flux rate and are expressed as the mean ± SEM of 8 cell layers per condition. **P < 0.01, ***P < 0.001 vs cobalt treated control knockdown. (Student’s t test, two-tailed)

If the effect of Co^++^ on transepithelial leak was being mediated by the well-described increase in HIF-1α levels caused by Co^++^ inhibition of HIF-1α breakdown, we reasoned that this would in fact be due to downstream effects of the transcriptional regulation exerted by increased levels of the HIF-1α/HIF-1β heterodimer, HIF-1. Therefore a pronounced drawdown of HIF-1β levels in the cell, prior to a stabilization or increase in HIF-1α levels caused by Co^++^, would result in a diminished effect on transepithelial permeability because of resultant lower levels of the HIF-1 heterodimer. As shown in Fig. 7, exposure of a HIF-1β knockdown variant of CaCo-2 (described in Saeedi et al. [12]) to 125 μM Co^++^ did in fact result in a sharply, significantly diminished response to Co^++^ relative to its transfection-control cell layers, with regard to both R_t_ and J_m_ as indicators of increased leak. We observed that this difference between the HIF-1β knockdown and its transfection control, with regard to effect of Co^++^, was apparent when 3-day-post-seeding cell layers were used, but was not observable when 7-day cell layers were used. In 7-day cell layers, Co^++^ caused similar relative increases in J_m_ and decreases in R_t_ when comparing the HIF-1β knockdown to its transfection control culture.

## Discussion

On a cursory review of the biomedical literature regarding the effect of HIF-1α on tight junctional permeability/barrier function, there seems to be an apparent contradiction. A very large body of mostly endothelial literature suggests that stabilization of (increased) HIF-1α levels induce increased leakiness in cellular barriers, an outcome with an array of pathophysiological implications (reviewed in Engelhardt et al. [8]). An excellent example of this literature is the study by Yan et al. [13] showing that the hyperglycemic induction of transendothelial (TJ) leak is mediated at least in part by HIF-1α. In fact, cobalt chloride stabilization of HIF-1α was shown to be one means of inducing endothelial barrier leak in this model. Yeh et al. [14] observed that pharmacologic inhibition of hypoxia-induced elevation of HIF-1α reduced the tight junctional disruption and barrier leak that ensued. This barrier-compromising action of HIF-1α is in fact not limited to reports using endothelial models. Cao et al. [15] report that the barrier-protective effects of berberine correlated with a suppression of HIF-1α activation in CaCo-2 epithelial cell layers.

On the other hand there is also compelling evidence in the literature that hypoxia and chemical hypoxia-mediated increases in HIF-1α levels can be supportive of barrier function. It is noteworthy here that this evidence of a barrier-friendly role for HIF-1 occurs in the context of barriers that have been already compromised in protocols mimicking the colitis condition. Karhausen et al. [16] noted in hapten-induced murine colitis, that decreased HIF-1 expression associated with more severe clinical symptoms regarding colon length, weight loss, etc. In dextran-sodium sulfate-induced murine colitis, the hydroxylase inhibitor, DMOG, was found to be protective regarding similar parameters [17]. In TNBS-induced murine colitis, another prolyl hydroxylase inhibitor stabilized HIF-1α levels and afforded protection in a similar manner [18]. Intestinal Trefoil Factor, one of many genes whose transcription can be upregulated by HIF-1, was found to reduce the barrier compromise induced by hypoxic conditions for CaCo-2 cell layers [19]. These findings regarding protective actions of HIF-1 in the context of hypoxia and colitis have been recently reviewed [9].

Our own data, obtained using the procedures we have detailed, support a barrier-compromising role for HIF-1. Three different prolyl hydroxylase inhibitors (Co^++^, DMOG, CPX) all increased transepithelial leak. The leak correlated with increased HIF-1α levels. Leak was reduced when HIF-1β levels were lowered, reducing the cells’ ability to form the needed HIF-1 complex. Increased leak was demonstrated by both significant decreases in R_t_ and significant increases in J_m_, and correlated with modified levels of integral TJ proteins, namely occludin and claudin-5. The leakiness was seemingly not attributable to induced cell death. This interpretation is supported by the size selectivity of the induced leak.

Additional work clearly still needs to be performed to develop a connection between increased HIF-1 levels and increased barrier leak in functional CaCo-2 cell layers. First, immunolocalization of increased HIF-1 specifically in the nucleus needs to be performed. Secondly, it would be very informative to analyze for HIF-1-mediated changes in transcription generally in these cells by performing chip assays focused on HIF-1 binding to various promoters.

We caution against over-interpretation of the TJ protein data presented here, wherein one might conclude e.g. that increased claudin-5 levels are not consistent with decreased barrier function. First, these are whole cell lysates being analyzed, and we therefore don’t know the contribution of cytosolic claudin-5. Secondly, it is not simply the level of claudin-5 (or any single claudin) that can predict the robustness of TJ barrier function. The heterotypic interactions of claudin-5 with other claudins — possibly also in play with cobalt treatment — may be even more important. Therefore it may be changes in ratios of claudin pairs that may be far more important in predicting or explaining TJ permeability changes than are the changes in any specific individual claudin. Lastly, it should be noted that CaCo-2 has many other TJ proteins than claudins −5, −7 and occludin. This is an incomplete presentation meant merely to show that HIF-1 modulation is modifying the TJ complex.

The involvement of HIF-1 in this action of Co^++^ that is presented here is supported by 3 pieces of evidence: 1) the increased levels of HIF-1α after Co^++^ exposure, a typical effect of Co^++^ in cultured cells [20, 21]; 2) the barrier compromise brought about by other agents of chemical hypoxia; and 3) the reduced effect of Co^++^ on barrier function in a CaCo-2 cell layer containing reduced levels of HIF-1β.

It is true that whereas HIF-1β knockdown is near complete, Co^++^ effects on barrier function were diminished but still apparent. One could reason that if Co^++^ action on barrier function is mediated through HIF-1 complex, the lack of the HIF-1β subunit should preclude an effect of Co^++^ on barrier function unlike the still significant effect that is shown here in Fig. 7. However Co^++^ has cellular effects other than the elevation of HIF-1α mediated by prolyl hydroxylase inhibition. Cobalt inhibition of the microsomal cytochrome P450 system and protein turnover in the cell is one example of a known effect of cobalt that could still be impacting the TJ complex in the absence of a HIF-1 elevation [22].

The peculiar polarity manifested in the action of Co^++^ on CaCo-2 BBe barrier function (Fig. 3) may simply be a result of polarized distribution of Co^++^ transporters in differentiated CaCo-2 cells, with greater Co^++^ entry into the cell across the basal-lateral membrane. This exact information regarding polar distribution of Co^++^ transporters in intestinal enterocytes or CaCo-2 cells is currently unknown.

Our finding that the state of differentiation of the CaCo-2 BBe cell population weighs significantly on the action of Co^++^ on barrier function (Fig. 6) may shed light on why HIF-1 can be viewed as barrier-unfriendly in some studies by certain research groups, while being barrier-friendly in others. We clearly observe that Co^++^ is unable to affect the barrier function of fully differentiated CaCo-2 BBe cell layers (21-day-post-seeding), yet has pronounced (negative) effects on the barrier function of less-differentiated cell layers (3-day-post-seeding). Therefore, the state of differentiation of a given cell culture or tissue model may matter tremendously in the exact effect observed. One should however note that the greater disruptive effect of Co^++^ on the barrier function of the less-differentiated, 3-day, cell layers — compared to the effect on the more differentiated, 21-day, cell layers (Fig. 6) — runs counter to the apparent barrier-supportive effect of HIF-1α in colitis models. In colitis models, the already wounded and disrupted gastrointestinal barrier should be less differentiated than its healthy counterpart mucosal barrier. However, there is more to the colitis state than simply a less-differentiated epithelial layer, and these other factors (inflammatory mediators, altered vascularization, etc.) could play significant roles in why HIF-1α could be barrier-supportive here.

The molecular mechanism(s) why cobalt action on 3-day cell layers is greater than on the more fully differentiated (21-day) cell layers could have many possible explanations. These could range from a different disposition of cobalt transporters, to different intracellular levels of HIF-1α or -β, to differentiation-dependent changes in junctional proteins or junctional-associated proteins. In addition, future work should also examine whether cobalt affects the cell cycling/division of CaCo-2 BBe cells, since such an effect would be disproportionately on the 3-day cell layers, as opposed to the 21-day cell layers, which are fully differentiated and likely not in the cell cycle.

In addition, it should be considered that there are a host of other factors that can impact not only HIF-1α and HIF-1 levels, but also the levels of downstream “players” such as VEGF that may themselves be directly impacting barrier function. This can be not only the rates of glycolysis and glucose concentrations in the cellular model [13, 23], but also the myriad factors that may impact the level of activation of the various PKC isoforms in the cell [24]. We feel it would be prudent to approach the issue in the biomedical literature from the perspective that both sets of studies are valid — those showing HIF-1 to positively as well as negatively affect barrier function. That being the case, it relies on future research to parse out the differences in methodologies, experimental conditions and cellular models that would result in these qualitative differences. Increased understanding of the mechanisms by which HIF-1 regulates barrier function will result.

## Conclusion

Exposure of CaCo-2 BBe cell monolayers to cobalt chloride resulted in elevated HIF-1α as well as increased transepithelial leak manifested by a decrease in transepithelial electrical resistance and an increase in the paracellular diffusion of D-mannitol. Along with the increased leak, we observed changes in TJ protein levels — decreased occludin and increased claudin-5 — that, accompanied by size selectivity of the leak, indicates an effect of cobalt on tight junctional permeability. The fact that this barrier compromise was seen using alternative inhibitors of HIF-1α breakdown, CPX and DMOG, and that a reduced effect of cobalt treatment was seen in a HIF-1β knockdown, both point to a role for HIF-1 in the impaired epithelial barrier function.

## Additional files

Additional file 1: Representative western immunoblot probed for Claudin-5, showing effect of cobalt exposure of CACO-2 cell layers. (TIFF 1975 kb)

Additional file 2: Representative western immunoblot probed for Claudin-7, showing effect of cobalt exposure of CACO-2 cell layers. (TIFF 2624 kb)

Additional file 3: Representative western immunoblot probed for HIF-1a, showing effect of cobalt exposure of CACO-2 cell layers. (TIFF 2014 kb)

Additional file 4: Representative western immunoblot probed for HIF-1b, comparing CACO-2 control cell layers vs CACO-2 HIF-1b knockdown cell layers. (TIFF 1975 kb)

Additional file 5: Representative western immunoblot probed for Occludin, showing effect of cobalt exposure of CACO-2 cell layers. (TIFF 2080 kb)

## Abbreviations

CPX: Ciclopirox olamine
DMOG: Dimethyloxalylglycine
HIF1: Hypoxia inducible factor-1
J_m_: Transepithelial mannitol flux
LSC: Liquid scintillation counting
PAGE: Polyacrylamide gel electrophoresis
PBS: Phosphate-buffered saline
RT: Room temperature
Rt: Transepithelial electrical resistance
SEM: Standard error of the mean
TJ: Tight junction
