# Supplementary figures and images for: Cobalt chloride compromises transepithelial barrier properties of CaCo-2 BBe human gastrointestinal epithelial cell layers
Source: BMC Gastroenterol. 2018 Jan 5;18:2. doi: 10.1186/s12876-017-0731-5 (PMC5756372; doi:10.1186/s12876-017-0731-5)

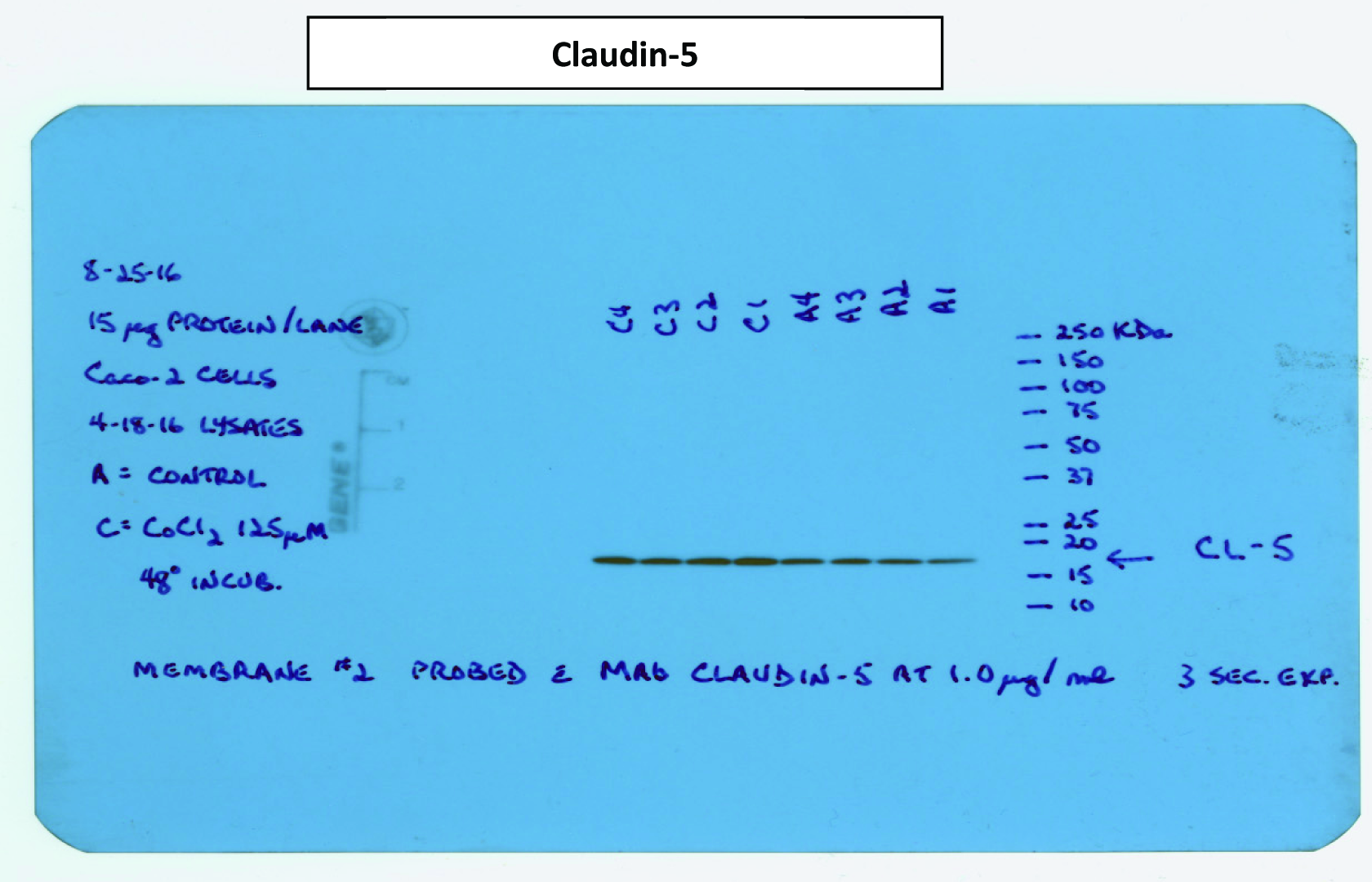

Supplement: Supplementary file 1 — Representative western immunoblot probed for Claudin-5, showing effect of cobalt exposure of CACO-2 cell layers. (TIFF 1975 kb) [file 12876_2017_731_MOESM1_ESM.tif]

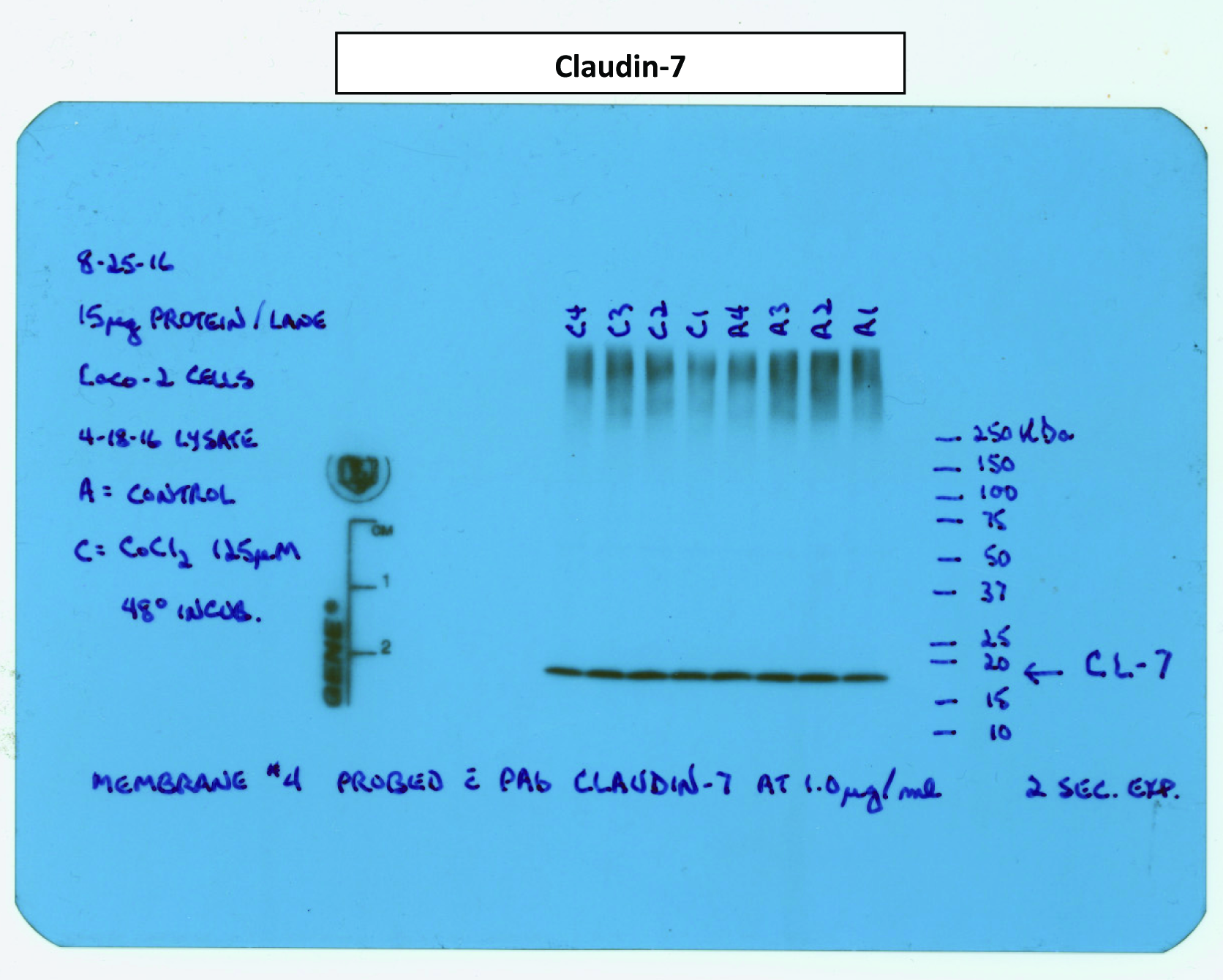

Supplement: Supplementary file 2 — Representative western immunoblot probed for Claudin-7, showing effect of cobalt exposure of CACO-2 cell layers. (TIFF 2624 kb) [file 12876_2017_731_MOESM2_ESM.tif]

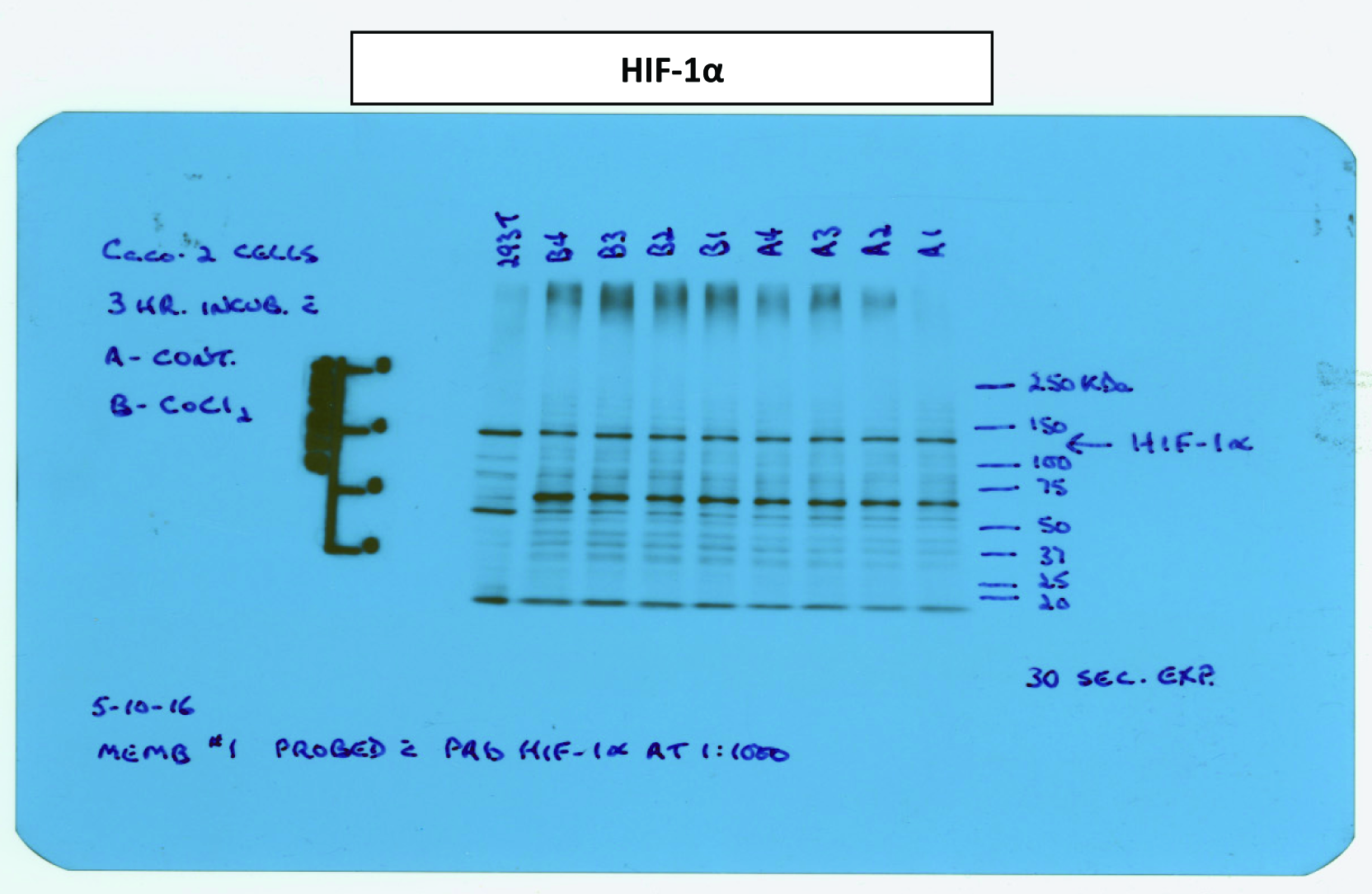

Supplement: Supplementary file 3 — Representative western immunoblot probed for HIF-1a, showing effect of cobalt exposure of CACO-2 cell layers. (TIFF 2014 kb) [file 12876_2017_731_MOESM3_ESM.tif]

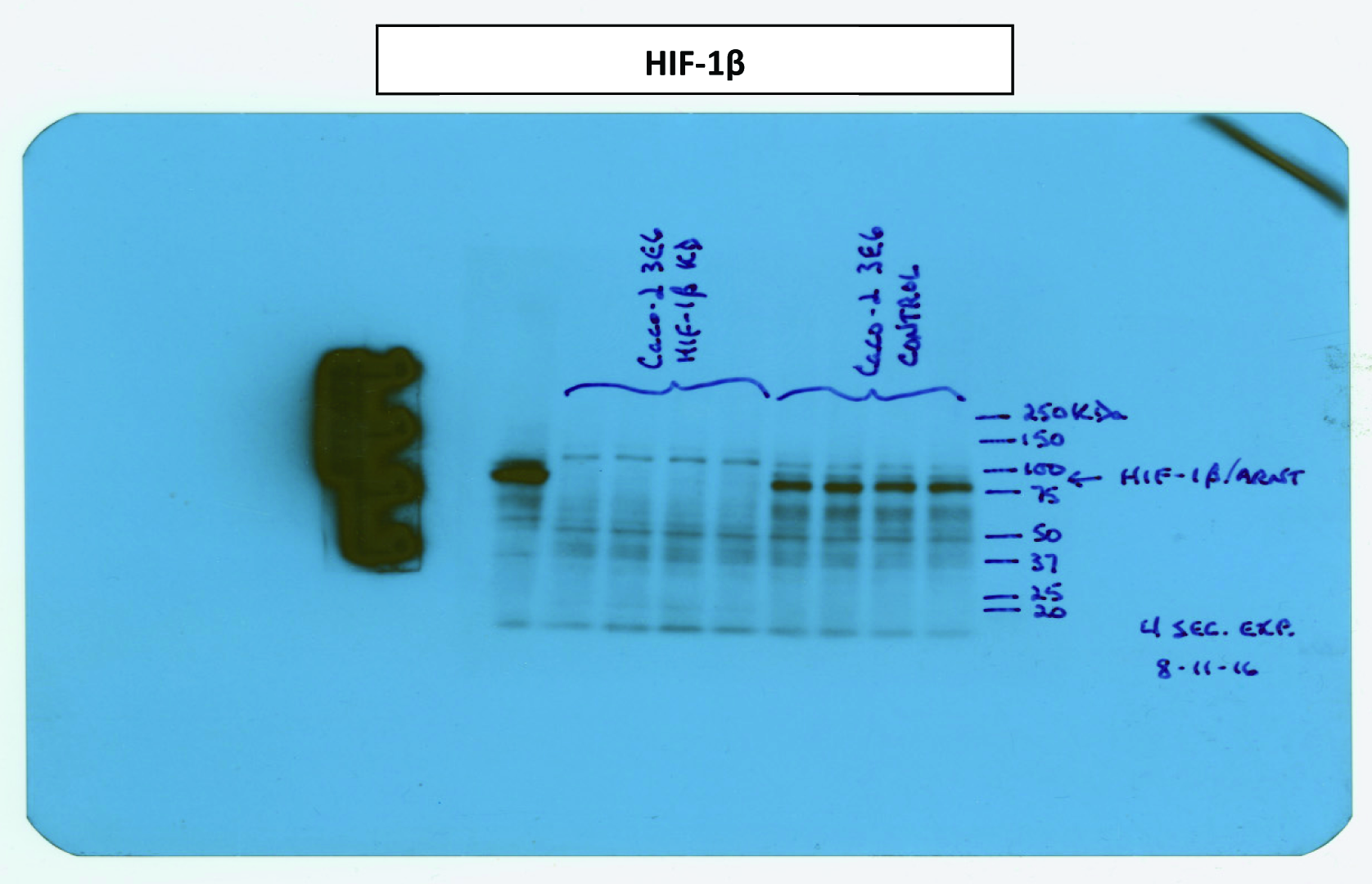

Supplement: Supplementary file 4 — Representative western immunoblot probed for HIF-1b, comparing CACO-2 control cell layers vs CACO-2 HIF-1b knockdown cell layers. (TIFF 1975 kb) [file 12876_2017_731_MOESM4_ESM.tif]

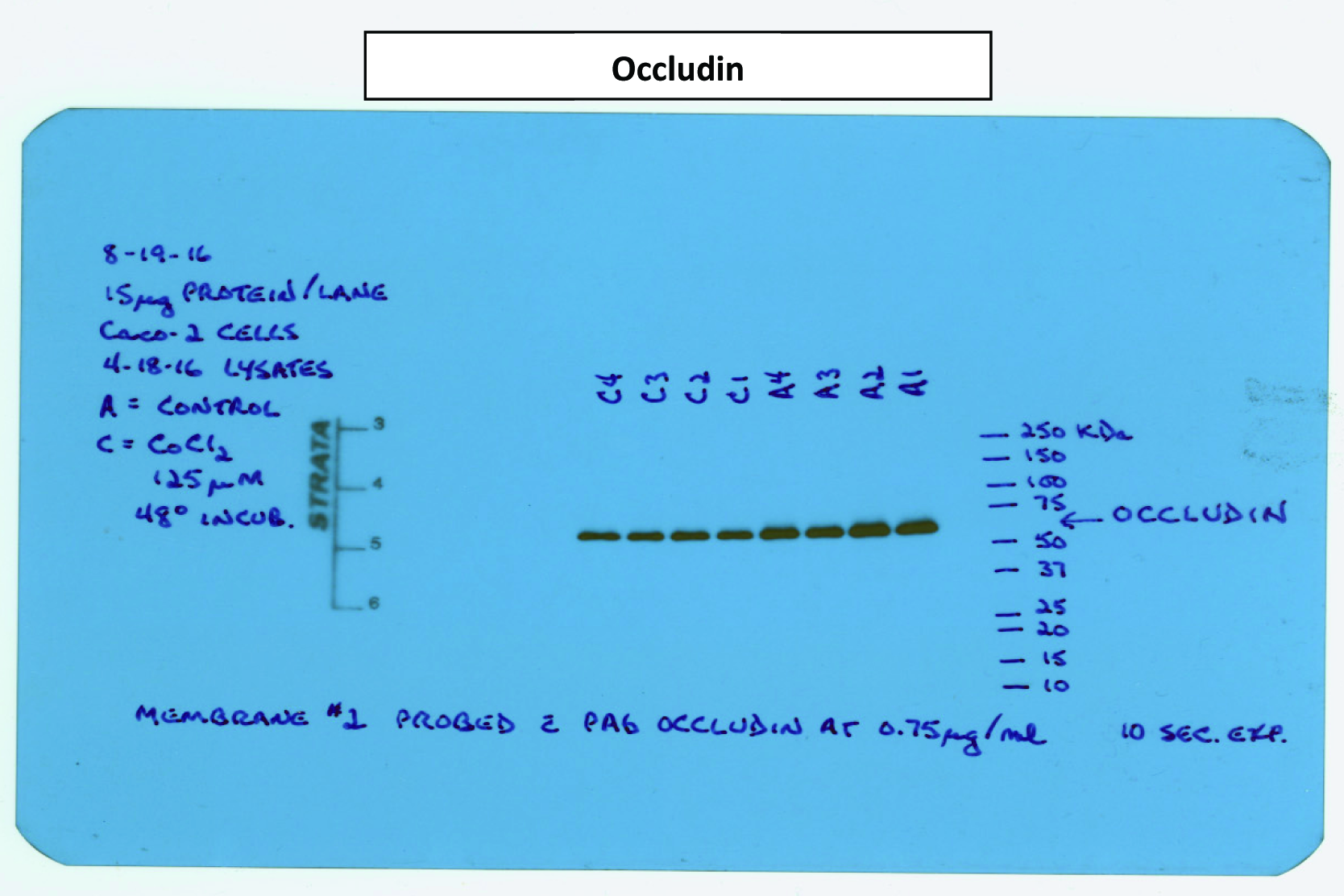

Supplement: Supplementary file 5 — Representative western immunoblot probed for Occludin, showing effect of cobalt exposure of CACO-2 cell layers. (TIFF 2080 kb) [file 12876_2017_731_MOESM5_ESM.tif]
